# Reply: Lipid Management in the Post-Statin Era: FDA Indications for Bempedoic Acid

**DOI:** 10.1016/j.jacadv.2025.102173

**Published:** 2025-09-18

**Authors:** Jared A. Spitz

**Affiliations:** Inova Schar Heart and Vascular, Inova Health System, Falls Church, Virginia, USA

We thank the authors for their kind words on our recent paper.^1^^,^^2^ We appreciate their update to the changes that occurred with the U.S. Food and Drug Administration label regarding bempedoic acid. We have made changes to Table 1 to reflect these changes in FDA labeling as well as the column on common usage/notes.

**Table 1:** Lipid Lowering Therapies^a^

Drug	Mechanism of Action	FDA Indications^b^	Common Usage/Notes	Lipid/Lipoprotein and Biomarker Changes	Reduction in Major Adverse Cardiovascular Events	Adverse Drug Effects
Statins	Inhibit HMG-CoA reductase	1. Primary prevention of ASCVD in those with increased cardiovascular risk 2. Established ASCVD 3. LDL-C reduction in primary hyperlipidemia 4. LDL-C reduction in heterozygous familial hypercholesterolemia 5. LDL-C reduction in homozygous familial hypercholesterolemia 6. Triglyceride reduction in hypertriglyceridemia and familial dysbetalipoproteinemia	1. First line lipid lowering therapy 2. Primary and secondary prevention indications 3. Cardiovascular outcome trials all on background statin therapy (except CLEAR OUTCOMES for bempedoic acid)	1. LDL-C reduction:≥50% for high intensity; 30%-49% for moderate intensity 2. Modest triglyceride reduction 3. Modest HDL-C increase 4. Reduction in hs-CRP	22% reduction for every 38 mg/dL LDL-C reduction	1. Statin associated muscle symptoms 2. Elevated transaminases 3. Dysglycemia with risk for new-onset diabetes
Ezetimibe	Cholesterol absorption inhibitor	1. Adjunct therapy for primary hyperlipidemia (eg, HeFH) 2. Pediatric HeFH 3. Adult and pediatric HoFH 4. Homozygous sitosterolemia 5. In combination with fenofibrate to reduce LDL-C in mixed hyperlipidemia	1. Primarily used as adjunctive medication for additional LDL-C lowering when patient above treatment threshold	1. ∼15%-20% LDL-C reduction 2. Modest triglyceride reduction 3. Modest HDL-C increase	∼6% reduction	1. Diarrhea 2. Arthralgias
Bile Acid Sequestrants	Bind intestinal bile acids	1. Adjunct to diet for primary hyperlipidemia (including HeFH)	1. Rarely used in routine lipid management 2. Safe for lipid management during pregnancy	1. ∼15%-30% LDL-C reduction 2. Can cause marked hypertriglyceridemia 3. Can low hemoglobin A1c	∼19% reduction	1. Gastrointestinal side effects 2. Contraindicated with hypertriglyceridemia
PCSK9 mAb (Evolocumab and Alirocumab)	Humanized monoclonal antibodies against PCSK9	Evolocumab: 1. Established ASCVD 2. Adjunct therapy for primary hyperlipidemia (eg, HeFH) 3. Pediatric HeFH 4. Adult and pediatric HoFH Alirocumab: 5. Established ASCVD 6. Adjunct therapy for primary hyperlipidemia (eg, HeFH)	1. Adjunct therapy in ASCVD 2. Adjunct therapy in HeFH 3. Adjunct therapy for additional LDL-C lowering when not at threshold per guidelines 4. Often used in statin intolerant individuals 5. Evolucumab and alirocumab often used interchangeably	1. ∼50%-60% reduction in LDL-C 2. Triglyceride reduction 3. ∼20%-30% reduction in Lp(a) 4. No hs-CRP reduction	∼15% reduction	1. Injection site reaction 2. Nasopharyngitis 3. Influenza like symptoms 4. Back pain (evolocumab)
Inclisiran	Small interfering RNA inhibitor of PCSK9	1. Established ASCVD 2. Adjunct therapy for primary hyperlipidemia (eg, HeFH) 3. High risk primary prevention above LDL-C threshold	1. Adjunct therapy in ASCVD 2. Adjunct therapy in HeFH 3. Adjunct therapy for additional LDL-C lowering when not at threshold per guidelines 4. Often used in statin intolerant individuals 5. Long-term cardiovascular outcome trials pending	1. ∼50% time averaged reduction in LDL-C 2. Triglyceride reduction 3. ∼20%-30% reduction in Lp(a) 4. No hs-CRP reduction	No cardiovascular outcome available (pending)	1. Injection site reactions 2. Arthralgias
Bempedoic Acid^2^	ATP-citrate lyase inhibitor	1. Established ASCVD 2. High-risk for ASCVD but without established disease 3. As an adjunct to diet alone or in combination with other lipid lowering medications for primary hyperlipidemia (e.g. HeFH)	1. Often used in statin intolerant individuals and as adjunct 2. Established ASCVD and high-risk for ASCVD 3. Adjunct therapy in HeFH	1. LDL-C reduction: ∼18% as monotherapy; ∼38% with fixed dose combination with ezetimibe; in combination with ezetimibe, ∼38% lowering 2. No reduction in Lp(a) 3. Reduction in hs-CRP	∼13% reduction	1. Hyperuricemia and gout 2. Tendon rupture: seen in those on high intensity statin, those with prior tendon injury, those taking corticosteroids and fluoroquinolone 3. Cholelithiasis
Icosapentethyl	Not established^c^	1. Adjunct to maximally tolerated statin to reduce ASCVD risk in those with TG ≥ 150 mg/dL and established ASCVD or diabetes and additional cardiovascular risk factors 2. As an adjunct to diet in severe hypertriglyceridemia ≥500 mg/dL	1. Only prescription fish oil shown to reduce cardiovascular outcomes after optimizing LDL-C lowering 2. Unknown if TG lowering with this medication reduces pancreatitis	1. ∼15%-20% reduction in triglycerides 2. Dramatic reduction in hs-CRP 3. Minimal reduction in LDL-C, apolipoprotein B (event reduction greater than expected for LDL-C lowering)	∼25% reduction	1. Increased risk of atrial fibrillation or atrial flutter (3% vs 2% in placebo group)-most common in those with history of atrial tachyarrhythmia 2. Increased risk of bleeding (12% vs 10% in placebo group; 3% serious bleeding)-greatest risk in those taking anti platelet or anticoagulant therapy

ASCVD = atherosclerotic cardiovascular disease; ATP = adenosine triphosphate; FDA = Food and Drug Administration; HDL-C = high-density lipoprotein cholesterol; HeFH = heterozygous familial hypercholesterolemia; hs-CRP = high-sensitivity C reactive protein; HoFH = homozygous familial hypercholesterolemia; HMG-CoA = 3-hydroxy-3-methylglutaryl coenzyme A; LDL-C = low-density lipoprotein cholesterol; Lp(a) = lipoprotein (a); mAb = monoclonal antibody; PCSK9 = proprotein convertase subtilisin kexin type 9; TG = triglycerides.

a This table is limited to those medications in common usage for treatment of hyperlipidemia for primary and secondary prevention. Medications used exclusively for homozygous familial hypercholesterolemia (lomitapide, evinacumab) or that are still in clinical trial (eg, for Lp(a) elevation and severe hypertriglyceridemia) are not included.

b Different statins may have slightly varied FDA indications based on clinical trial data. However, this represents a composite of FDA indications for the generically available statins; specific FDA indications should be consulted as necessary.

c Believed to reduce hepatic triglyceride synthesis and packaging and/or increase triglyceride clearance from circulation.
